# European community pharmacists practice in tackling influenza

**DOI:** 10.1016/j.rcsop.2024.100447

**Published:** 2024-04-22

**Authors:** Marleen Haems, Mauro Lanzilotto, Andrea Mandelli, Hélder Mota-Filipe, Ema Paulino, Beata Plewka, Olivier Rozaire, Jens Zeiger

**Affiliations:** a Koninklijk Oost-Vlaams Apothekersgild (KOVAG, Royal Society of Pharmacists of East Flanders), Brouwerijstraat 1, 9031 Ghent, Belgium; b Federazione Nazionale dei Titolari di Farmacia Italiani (Federfarma, National Federation of the Italian Pharmacy Owner), Via Emanuele Filiberto 190, 00185 Rome, Italy; c Federazione degli Ordini dei Farmacisti Italiani (FOFI, Federation of the Orders of Italian Pharmacists), Via Palestro 75, 00185 Rome, Italy; d Faculty of Pharmacy of the Universidade de Lisboa (FFUL), Avenida Professor Gama Pinto, 1649-003 Lisbon, Portugal; e Associação Nacional das Farmácias (ANF, National Association of Pharmacies), Rua Marechal Saldanha 1, 1249-069 Lisbon, Portugal; f Pharmacy Practice Division, Chair and Department of Pharmaceutical Technology, Poznan University of Medical Sciences, 6 Grunwaldzka Street, 60-780 Poznan, Poland; g Union Régionale des Professionnels de Santé Pharmaciens Auvergne Rhône Alpes (URPS AuRA, Regional Union of the Healthcare Professionals, Pharmacists, Auvergne Rhône Alpes), rue Garibaldi 194B, 69003 Lyon, France; h Marketing Verein Deutscher Apotheker (MVDA, Marketing Association of German Pharmacists), Emil-Hoffmann-Straße 1a, 50996 Cologne, Germany

**Keywords:** Influenza, COVID-19, Vaccination, Pharmacist, Pharmacy, Public health

## Abstract

**Background:**

In many European countries, flu vaccination coverage rates are below the 75% target. During the COVID-19 pandemic, many pharmacists around Europe were involved as vaccine administrators and demonstrated positive results in improving vaccine uptake. This paper explores the challenges, accomplishments, and best practices of various European pharmacists' associations in administering vaccines and positively contributing to public health.

**Methods:**

Eight pharmacists representing various associations from different countries across Europe (Italy, Belgium, Poland, Portugal, France, and Germany) convened to discuss their role as vaccination providers, the advantages, and strategies for improvement, and to identify barriers and gaps in the vaccination administration process, especially focusing on the administration of seasonal flu vaccines.

**Results:**

Currently, 15 European countries allow community pharmacists to dispense and administer flu vaccines. Among the ones that attended the meeting, Portugal initiated the flu immunization program at the pharmacy earliest, before the COVID era, but in other countries, the process started only in the last couple of years. Initial hesitancy and reluctance by other HCPs or institutions were overcome as the pilot projects showed positive and cost-effective public health results. Today, pharmacists are considered crucial professional figures to provide immunization services against COVID-19, the flu, and other vaccine-preventable diseases, and pursue important public health goals.

Key takeaways to enhance the pharmacist's role in providing immunization services against vaccine-preventable diseases include improving interaction with policymakers and the public, generating real-world evidence highlighting public health benefits, and ensuring ongoing professional education and training for pharmacists.

**Conclusion:**

Vaccinating pharmacists are gaining recognition of their role and the benefits derived from their broader involvement in the healthcare system, including immunization programs. Further efforts are needed in each country for an adequate recognition of the profession and a broader utilization of pharmacy services to exploit the benefit of immunization, especially against the flu.

## 1 Introduction

Globally, Influenza affects 10 to 30% of the human population every year, causing 250,000 to 500,000 deaths due to its associated complications.^1^ The European Centre for Disease Prevention and Control (ECDC) estimated fifty million symptomatic cases of influenza including 70,000 deaths in the European Union (EU) alone each year.^2^

Seasonal vaccination is a highly effective preventive strategy against influenza. Recent studies reported that flu vaccination reduces the risk of life-threatening influenza illness by 75% in children^3^ and reduces the risk of ICU admission by 26% and deaths by 31% in vaccinated adults.^4^

Despite such benefits, the coverage rates in the EU are below the target of 75%, with coverage rates in some countries below 20%.^5^ Coordination of existing services and implementation of new vaccination strategies may improve the coverage rates. A recent study involving pharmacists from 16 European countries demonstrated their diverse contributions during the COVID-19 situation and highlighted their readiness to be involved in the vaccination campaign as vaccine administrators.^6^ Indeed, since its publication, various improvements have been implemented across Europe, consolidating the active role of pharmacists in dispensing and administering vaccination against COVID-19, and expanding their responsibilities to other immunization campaigns, such as against the flu.

There are 180,000 pharmacies in Europe serving 3245 citizens on average. Sixty percent of EU citizens can reach the nearest pharmacy within a 5 min' walk and pharmacies are the first point of contact between patients and the healthcare system. European pharmacies offer multiple services such as generic substitution, pharmacovigilance, preparing tailored medications, providing medicines during out-of-hours (night services), dose/device administration aid, delivering/administering vaccination, and screening and referral.^7^

**Fig. 1:**
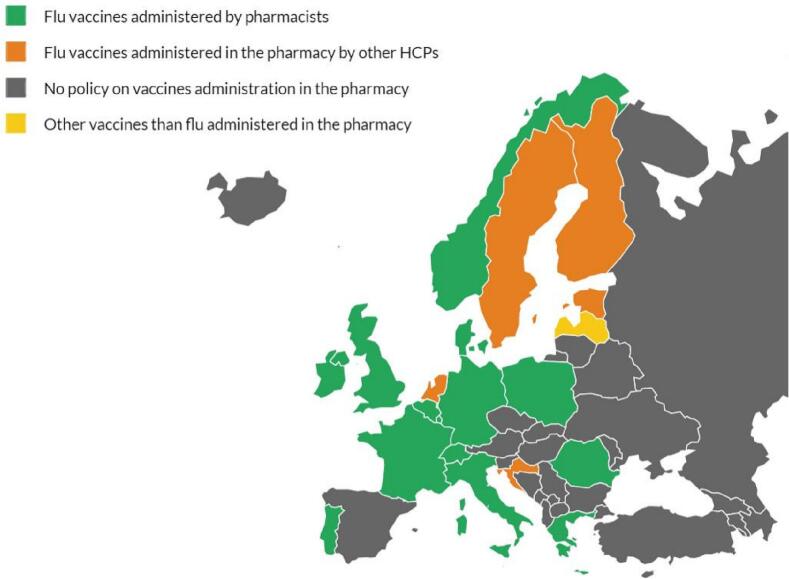
Status of Vaccinating Pharmacists against the flu in Europe.

In many European countries, community pharmacists are allowed to dispense and administer flu vaccines, including the supply of flu vaccines to at-risk groups without the prior need for a prescription (Fig. 1).^8^ This led to a growth in flu vaccine uptake. For instance, a pilot program in Portugal during the 2008/2009 flu season resulted in more than 150,00 to receive their influenza vaccine in the pharmacy. This accounted for more than one-third (36.4%) of the total number of vaccines administered during the season. Vaccination coverage was estimated at 50.4% among users aged 65 years or over, with the contribution of pharmacies in this subgroup being between 4.4 and 10.8%.^9^ In Ireland, following the introduction of flu vaccination services in pharmacies in 2011, the national flu vaccine delivery has increased by 59.5%, while GP flu vaccine delivery has seen an increase of 27%.^10^ Similar research conducted in Canada found that 28% of those vaccinated would not have been without the pharmacy-based service, and 21% of these were at-risk patients.^11^This highlights the positive impact that pharmacy-led vaccination can have to improve overall vaccination rates and reach the 75% target set by the WHO.

When active, pharmacies ensured the continuity of immunization services during and after the pandemic. For instance, data collected in France related to COVID-19 vaccination reported that pharmacists were the major contributors of vaccination compared to all other healthcare professionals: considering the peak week of vaccination (19 dec 2021), pharmacists delivered 1,268,490 doses over a total of 2,346,990 doses, accounting for 54% of administration for that week.^12^

Different studies conducted in the UK highlighted the positive public health impact of including community pharmacies in the immunization campaigns: they can provide additional capacity,^13^ they are open longer and have a broader distribution, including remote locations, and are accessible to hard to reach patients^14^ and they can reach out to patients not previously vaccinated by GPs.^15^Further, community pharmacists identify and remind the target groups about their vaccination schedule, advocate several vaccine awareness campaigns, and provide evidence-based, unbiased, and balanced information on the benefits of vaccination (patient counseling sessions). This can lead to multiple advantages across the different stakeholders in the healthcare system.^16^

A previous systematic review showed that vaccination campaigns led by pharmacists are widely accepted by patients, which helped in improving access and vaccination rates.^17^ Another systematic review reported an increase in vaccine coverage rates when pharmacists engaged in the immunization process regardless of role (educator/facilitator/administrator) or vaccine administered.^18^ Further, the pharmacists contributions in increasing the vaccination rates against flu is highlighted by a review conducted by Rahim et al. (2023).^19^ The findings demonstrated an enhanced immunization uptake rates up to 51% irrespective of the pharmacists' specific interventions and the frequency of immunization doubled when pharmacists acted specifically as advocators.

However, barriers at the political and organizational level may prevent pharmacists from taking part in vaccination campaigns.^17^

To date, there is little data available describing the involvement and the impact of pharmacists as flu vaccine administrators at the European level, especially in the post-pandemic period. This consensus paper aims at providing an updated picture of the role of pharmacists in the immunization campaigns, especially regarding influenza prevention, describes the challenges pharmacists face, the accomplishments they have achieved, and provides a platform to share the best practices they follow in administering flu vaccines in their respective countries.

## 2 Methods

A group of pharmacists (eight), representatives of pharmacists, pharmacy associations and professional regulatory organizations from different countries across Europe, have taken part in a virtual advisory board meeting conducted by Viatris on May 2023. The group was convened to discuss the role of pharmacists as vaccination providers, the advantages of having pharmacists as vaccination providers, strategies to improve their role, and to identify barriers and gaps to facilitate and improve the process.

The meeting was facilitated by a Viatris Global Medical Affairs representative, who had the responsibility to build and manage the agenda, present the summary of the qualitative literature search on the role of pharmacists in flu vaccination in Europe, define the form and collate feedback from the participants, and moderate the discussion session. Meeting minutes were taken, circulated among participants, and approved before finalizing the meeting report.

The participants should be practicing pharmacists in a European country with relevant roles in national pharmacists' associations or trade associations, or with an academic position. Participants were selected based on their experience in flu vaccination at the pharmacy level, or their role as national pharmacists' association representatives in promoting and implementing the model. For example, the meeting included pharmacists with long-term experience (Portugal or France, that started pilot programs before the pandemic), moderate experience (like Italy that initiated the vaccine administration during the pandemic) and pharmacists that have recent experience during or after the pandemic (i.e. Poland, Germany, Belgium).

Prior to the meeting, a qualitative literature search was conducted to gather information and data on the ‘role of pharmacists in flu vaccination in Europe’ focusing the period from April 2013 to April 2023. The participants also completed a form covering various aspects such as 1) Their role as vaccine administrators and the mandatory requirements, including details of the legislative framework for practice, 2) The category of general population eligible for vaccination at the pharmacy, along with information about access and reimbursement models, 3) Metrics involving number of pharmacies/ pharmacists, the percentage of sites and pharmacists providing vaccination, and number of vaccines administered in their respective country, 4) Achievements and barriers encountered during the implementation of the vaccination model and, 5) Timing and process for implementation. The data from the literature search and information provided by each participant was summarized in form of a presentation which was shared as a pre-read before the meeting. The same information and data were used to drive the discussion of the meeting and were presented as a narrative/best practices in the results section. The discussion section summarized the discussion in the roundtable on the discussion items 1 to 5 listed above.

Therefore, the information provided in this paper reflects the views of the pharmacists and the practices they follow in administering flu vaccines in their respective countries. In some cases, the regulatory and legislative framework has changed after the meeting, and the information was therefore updated accordingly.The discussion session was guided by a series of topics to explore: A) Regulatory/legislative framework; B) Mandatory training/certifications, Facilities and equipment; C) Evidence generation initatives; D) Guidelines and reccomendations for target groups.

## 3 Best practices

### 3.1 Italy

Italian pharmacists were fully involved in the vaccination campaign during the COVID-19 pandemic, primarily to shorten the patient's journey to get the COVID-19 vaccine starting from July 2021. Flu vaccine was added to the list of vaccines that can be administered at the pharmacy starting from flu season 2021/22.

The collaboration between public institutions, the Italian Federation of Pharmacists' Associations, the trade Associations, policymakers, and the political environment were crucial to assess and reinforce the role of pharmacists and community pharmacies in the delivery of proximity healthcare, especially in the vaccinal area.

The Yearly Report of Italian Pharmacy released by Federfarma in collaboration with the civic association Active Citizenship, showed that even before the pandemic, pharmacies already held a high reputation among the population, and were interested in being more involved in vaccination campaigns. The growing feeling of mutual trust between people and pharmacists made clear to the political environment that pharmacies were a precious hub for public healthcare.

During the COVID-19 pandemic, urgent actions were needed to facilitate the distribution of individual protection devices, performing rapid Covid-19 swabs, and then the vaccination campaign. The initial opposition from the healthcare professionals (HCPs) to the role of immunizing pharmacists was overcome by the need of a fast response to protect, build up the population immunity and to control the pandemic. Policymakers swiftly approved crucial regulations allowing a more active role of pharmacists in immunization and diagnostic areas (vaccines administration, swabs, and rapid blood tests). In this framework, the pandemic positively accelerated the de-hospitalization of various health procedures in Italy: HCPs working in the field had been allowed to take care of the patients more closely, providing relief to hospitals and releasing needed resources for in-hospital assistance, such as in ERs or ICUs. In this way, pharmacies were recognized as a key healthcare facility, already part of the national healthcare system, and able to connect with even the most rural and difficult to reach areas in the whole country.

Currently, more than 50,000 out of 76,000 community pharmacists (66%) in Italy are qualified for vaccination.^20^ The administration of vaccines is performed by qualified pharmacists, after certification of a specific training course and annual updates organized by the Superior Health Institute (Istituto Superiore di Sanità). Although pharmacists cannot formally prescribe, they can administer flu vaccines without a prescription. The anamnesis signed by a patient serves as a medical prescription.

Pharmacists can administer flu vaccines to all categories of patients except age groups below 18 years old, and people who are new to vaccination. Flu vaccines and administration procedures are fully reimbursed by the National Health Service (NHS) for the elderly, pregnant women, patients suffering from chronic medical conditions, health and police workers, veterinarians, and people working in close contact with animals. Each administrative Region can decide to enlarge the free vaccination scheme in case of an increased public health risk or if recommended by governing bodies.

Allowing pharmacists to take part in other immunization programs (other than flu) and to vaccinate people who were never vaccinated before are the next goals of pharmacy associations to improve coverage rates. The first achievement in this direction was attained when pharmacists signed an initiative to administer shingles vaccines in March 2023 and a pilot program started from 1st December 2023 in Marche Region.

### 3.2 France

In contrast to Italy, the initiative to administer flu vaccination started in 2017, as a pilot project, but only in a limited group. Later in 2018, the project expanded to vaccinate all adults who are eligible for influenza vaccination and by 2021, thanks to the dedication of the French Pharmacists Unions, pharmacists had the authorization to vaccinate all adults, including those not included in the guidelines. Currently, pharmacists can prescribe and administer all vaccines (including flu) for patients aged more than 11 years.

The requirements to be a vaccinating pharmacist in France include mandatory certified training and having a suitable place to practice. Vaccines are reimbursed with or without a medical prescription, by the public health system, or co-paid by private insurances.

Doctors and nurses were initially hesitant about supporting pharmacists to administer vaccines, but two factors contributed to change this opinion: first, the vast number of vaccines administered by pharmacists, accounting for a growth from zero to 21 million in 5 years (2016–2021) and more than 90% of French pharmacies active in vaccination; second, the relief that pharmacists can offer on the burden on the healthcare system, including the lack of medical personnels and their general work overload. The latest COVID-19 vaccine data reports that approximately 26 million of doses, 50% of the total number injections, from 2020 to 2022, have been done by pharmacists.^12^ Similar results have been obtained for flu: during the 2020–21 flu season, 87% of French pharmacies participated in the flu vaccination program, with more than one in three flu vaccination administered by pharmacists.^21^ Latest data about the vaccines prescriptions in pharmacy showed that approximately 50% of the pharmacists declared to have prescribed a vaccine to their patients, COVID-19 and flu accounting for 84% and 73% respectively, and 98% of pharmacists have proposed to administer a vaccine.^22^

### 3.3 Portugal

In Portugal, pharmacists have been allowed to vaccinate since 2007. The provision of this service initially faced opposition from the Nursing Society, however, based on the support from other HCPs and the public, pharmacists have been counseling and administering vaccines since then. Community pharmacists have been vaccinating people in Portugal with the vaccines which are not covered by the National Vaccination Programme. The service may be reimbursed by the Government or may be paid out-of-pocket by patients.

From 2023, community pharmacists can dispense and administer both flu vaccines and COVID-19 vaccines to all individuals over 60 years old, without a prescription, fully reimbursed by the Portuguese NHS. Reimbursement for vaccines and it's administration has changed over time. For the last flu season 2023–24, in the retail market, only standard dose vaccines were partially reimbursed; in the tender market, all the flu vaccines were fully reimbursed, according to the Risk groups recommendations from the Health Directorate (DGS). Some private companies, along with their Work Medicine physicians, are covering the vaccine costs for their employees. At pharmacies, under the umbrella of DGS recommendations, all people over 60 years old can receive the vaccine for free. The cost of the service is covered by the government.

Pharmacists can also administer vaccines in community pharmacies to HCPs who work at the pharmacy. The NHS, through primary healthcare centres, administers flu and COVID-19 vaccines to at risk patients with less than 60 years old, and to other HCPs. Considering this, an estimated 2.5 million people would be vaccinated against the flu, and around 70% would receive their injection at the pharmacy.^23^ From 2023, community pharmacists can also access the eVaccination bulletin, as well as register the vaccine administration in the national registry.

It is mandatory for pharmacists to be trained and obtain a specific Pharmaceutical Competence certification from the Portuguese Pharmaceutical Society (PPS) to administer injectable medicines and vaccines in community pharmacies. As of today, the Portuguese Pharmaceutical Society has about 7000 pharmacists who have been awarded a Pharmaceutical Competence certificate and are able to administer vaccines and other injectable medication.^24^

Nearly, 2500 out of 2921 (85.5%) of Portuguese pharmacies are active in administering vaccines.^25^^,^^26^ The increase in the number of participating pharmacies and the average number of flu vaccines administered in pharmacies are positive indicators of the successful impact of having pharmacists onboard in the vaccination program. Surveys reported 95% satisfaction with the service provided by the pharmacy and 99.5% satisfaction with the pharmacists who administered the vaccine.^27^

The next set of goals for the Portuguese pharmacist's and pharmacy associations include increasing free administration of other vaccines in partnership with the NHS, administering more vaccines without the need for a medical prescription, taking part in vaccination campaigns other than flu and COVID-19, and having at least one mandatory authorized vaccination provider per pharmacy.

### 3.4 Germany

In Germany, the legal provisions for a pharmacist to vaccinate have been in place since 2021. Pharmacists participated in the COVID-19 vaccination campaign and contributed to an increase in vaccination coverage rates. Even before that, there were regional pilot projects to allow pharmacists to vaccinate also against influenza. In autumn 2022, it became officially a regular service, meaning that all pharmacists who meet certain requirements can vaccinate against influenza.

To be a vaccine administrator in Germany, pharmacists must undergo a specific medical training (covering administration techniques and safety procedures) and must meet specific infra-structure requirements.

Pharmacists can, in principle, administer flu vaccine to any patients over the age of 18 years. Vaccination in accordance with the Standing Committee on Vaccination (STIKO) recommendations is covered by all health insurance funds; in addition, many health insurance funds also reimburse vaccination for other individuals.

Currently, less than 10% of German pharmacies are active in administering flu and COVID-19 vaccines.^28^

Some strategies proposed to improve vaccination coverage rates include providing vaccination during off-peak hours, while doctors are usually closed (i.e., Afternoons after 5 p.m., Wednesday and Friday afternoons and Saturdays), providing vaccinations to people who work long hours, focusing on people who do not have their own family doctor, and people interested in getting quick vaccinations without prior appointments.

### 3.5 Poland

In Poland, vaccination against influenza in pharmacies began in January 2022, pursuant to the Regulation of the Minister of Health, which states that pharmacists who are authorized to perform vaccinations against COVID-19 are also authorized to perform vaccinations against influenza. The trainings to obtain a certificate authorizing to perform the vaccinations took place in 2021 and has been resumed in November 2023.

Certified pharmacists can prescribe, qualify, and vaccinate all adults against influenza. From September 2023, eligible individuals (adults 18–65 years, children, elderly and pregnant women) can receive the vaccination at the pharmacy with reimbursement, but only if they present a prescription from a doctor. Prescriptions issued by pharmacists are not reimbursed.

During the pandemic, The National Health Fund financed the vaccination service from September 1, 2022, to March 31, 2023. Since November this year, it also finances the vaccination qualification process and the administration of the flu vaccine in a community pharmacy, but only in the case of vaccination of a person over 65 years of age. However, to purchase a vaccine with reimbursement, the patient must have a prescription from a doctor. Other patients can get vaccinated at a pharmacy, but they must pay for this service. There are more than 12,700 pharmacies of which approx. 1500 are able or will be able soon to administer vaccines.^29^ About 2.5 million COVID-19 vaccinations and 130,000 flu vaccinations have been administered in pharmacies between the beginning of the pandemic and the end of the flu season in March 2023.

Pharmacists, through professional organizations such as the Pharmaceutical Chamber, pointed out the unused potential of pharmacists in the context of vaccinations long before the pandemic, but only with the pandemic specific legislative actions were taken, removing obstacles such as the extreme delay in authorizing pharmacists to administer and prescribe flu vaccines. In particular, the cooperation between the association of the National Influenza Control Program, the Supreme Chamber of Pharmacy, and the Trade Union of Pharmacy Employees in establishing the “Coalition for vaccination in pharmacies”, allowed better interactions with politicians on the legislative changes needed to introduce the vaccination services to pharmacies. The role of pharmacists in vaccination is well described in the “Report – Pharmaceutical Care. Flu vaccinations in pharmacies”.^30^

An important goal for the future is to enable pharmacists to issue reimbursed prescriptions, which will encourage patients to use pharmacies as their vaccination points. Moreover, vaccination services at pharmacies should be reimbursed for all adults. In the current situation, every insured person can get vaccinated free of charge at a public health care clinic. However, in pharmacies it is only possible for people over 65 years of age.

### 3.6 Belgium

Since 2019, Belgian pharmacists were allowed to prescribe flu vaccines, but not administering them. After the pandemic, they were allowed to administer COVID-19 vaccine, and a new legislation was recently passed to include pharmacists in administering flu vaccines, with implementation as of October 1, 2023. It is a pilot project until 31 December 2023, with an evaluation afterwards. There is no difference in reimbursement if it is prescribed and performed by a doctor or a pharmacist. All patients belonging to the target group or asking for a flu vaccination can be vaccinated by the pharmacist, although it is recommended not to vaccinate children in pharmacy. Pharmacists make a registration for each administered vaccination in the appropriate regional digital systems.

More than 6200 out of the 9000 in Belgium pharmacies offered flu vaccination in 2023–24 flu season.^20^^,^^31^

The training consists of a total of 8 h training, 4 h of theory and 4 h of practice, including mandatory Basic Life Support training, valid for three years, after which it should be repeated. Moreover, the pharmacy rooms should also meet specific requirements to make the vaccine administration possible.

The biggest barrier in allowing pharmacists to both prescribe and administer flu vaccines was assigning the right place to the different types of vaccine administrators in the vaccination landscape. The solution lies in doctors continuing to do most of the vaccination, aided by nurses for persons with reduced mobility or at collectively organized vaccination events. Because of their unique accessibility, pharmacists complement them in reaching patients for whom the step to the doctor is too big and who do not use a home nurse either. In the COVID-19 vaccination, too, the Belgian pharmacists played a key role due to their unique accessibility. In addition, a large part of doctors is overburdened and consider the inclusion of pharmacists in vaccination programs as a welcome step.

The key indicators Belgium pharmacists consider measuring the impact of pharmacists as vaccine administrators include tracking the number of vaccinations, dispensation and prescription data of vaccines, and impact on vaccination rates as well. Counseling and raising awareness of the defined risk groups by sharing alerts are also important achievements of Belgian pharmacists.

During 2023–24 flu season, Belgium pharmacists delivered more than 3.8 million doses of flu vaccines, and directly administered nearly 15% of them (range 13.6–28.9% depending on the region). However, the impact of vaccination by pharmacists on vaccination rates in not yet known.^31^

## 4 Discussion

As described in previous study,^6^ the pharmacist's involvement in COVID-19 vaccination was crucial in the initial phases of the pandemic and included for instance education and training to HCPs and patients, facilitation of routine clinical services, vaccine logistic support and administration of COVID-19 vaccines, providing a preferential way to allow mass vaccination in the shortest time period. The positive experience gathered during the pandemic provides a robust background for extension of the pharmacist-led programs, while the efforts of different professional societies in Europe lead to relevant changes in legislative provisions to expand the scope of immunizing pharmacists beyond COVID-19 vaccination campaign, including also other vaccine preventable diseases such as the flu.

About 2 years later from the picture described by Paudyal in 2021,^6^ the legal framework for vaccinating pharmacists in community setting, especially regarding flu immunization programs, is still fragmented.

In countries such as Portugal, pharmacists have been administering flu vaccines since 2007, and the COVID-19 pandemic has reinforced and highlighted their role in the public health space. In the post-pandemic era, the professional figure of immunizing pharmacists has been confirmed for Italy, Germany and Switzerland, or newly introduced in Belgium. Vaccinating pharmacists are nowadays key players also in other countries not mentioned by the Paudyal paper,^6^ such as France, Denmark, Poland, Romania, Greece.

The roundtable led during the meeting was thus useful to share among the countries the key learnings, and the plans and to identify actions able to streamline the effective implementation of flu vaccine campaigns.

Previous publications have already highlighted the gaps, barriers and requirements needed to streamline the adoption of the immunizing pharmacists model: to mention, the need for a specific regulatory and legislative framework, the inclusion of the payor perspective over drug and procedure costs, the need for specific accreditation and continuous training, the need for an accessible service and for easy procedures to comply with the mandatory requirements and for record keeping.^6^^,^^32^

**Fig. 2:**
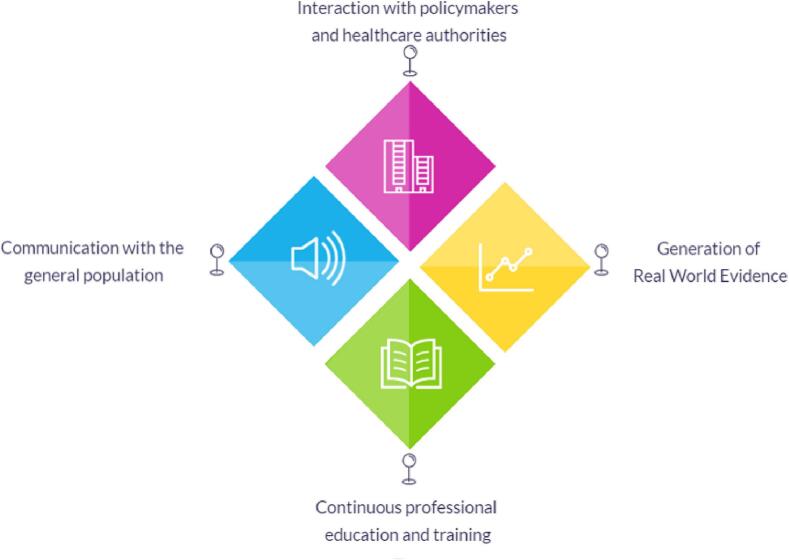
Key areas for improvement.

The following four topics have been identified (Fig. 2) and discussed as priorities, along with the respective actions and areas of improvement.

### 4.1 Lack of interaction between pharmacists and policymakers

Despite being identified also in previous studies,^6^ it occurs quite often that politicians/policymakers do not know or recognize the role of vaccinating pharmacists. There is a general lack of information about the effective delivery of vaccination programs by pharmacists. Pharmacists' associations may improve the collaboration among them and with the healthcare authorities at the country and EU level to influence the political arena in this respect. A broader inclusion of the view of different stakeholders, such as policymakers and other HCPs, is still an actual action that can be beneficial to delegate further roles to pharmacists with a view to promoting changes in relevant legal frameworks to allow to administer vaccines. With respect to this point, it is worth to mention the best practice cases of British Columbia and Nova Scotia, Canada, where the respective College of Pharmacists and Pharmacy Associations collaborated with multiple stakeholders, such as HCPs (pharmacists, physicians, nurses) and institutions, before authorizing pharmacists to vaccinate, leading to an effective and timely implementation of the legislative changes.^33^ Pharmacy and Pharmacists' associations should reinforce their connections with politicians and policymakers to establish a positive mindset around vaccinating pharmacists, reinforcing the role, and facilitating the transition to the new role.

### 4.2 Generation of effectiveness data on the activity of vaccinating pharmacists

As it became clear while preparing the pre-readings for the meeting and during the roundtable session, there is a lack of publicly accessible scientific data to show the public health benefits of vaccinating pharmacists, especially covering the European space in the post-pandemic period. For instance, an economic analysis of pharmacist-led flu vaccinations based on data collected in Ontario, Canada, and comparing two consecutive seasons, showed meaningful insights on the potential savings in direct healthcare costs and lost productivity associated with the increased coverage brought by pharmacists' vaccination.^34^ Therefore, more research should be undertaken to delve not only into patient satisfaction and acceptance of pharmacist services, but to assess the cost-effectiveness of such services as well. This would assist in promoting/expanding the role of pharmacists as vaccine administrators at national and international level and contributing to relief the economic pressure to the healthcare systems. Pharmacy and pharmacists' associations should support pharmacists in collecting and publishing real-world data and identify more indicators or methodology parameters that support the advocacy of the role of pharmacists in vaccination.

### 4.3 Continuing education and training of pharmacists

Ensuring that pharmacists are fit to recommend and administer vaccines is crucial. A recent study conducted among three pharmacy undergraduates in Germany showed that even a basic training, integrated in pharmacy curriculum, could significantly increase the level of the self-assessment and preparedness for routine pharmacy vaccination in the future.^35^

Additional training may assist pharmacists in identifying and vaccinate ‘risk groups’ such as pregnant women. In countries where pharmacists are not allowed to vaccinate risk groups, training may help them identify and encourage risk groups to get vaccinated from an HCP, thereby increasing vaccine coverage rates. In addition to training, conducting practice-based activities, and supporting pharmacists financially to take part in continuing medical education (CME) programs may help in improving pharmacists' confidence, knowledge, and skills. Low confidence levels can cause low vaccine recommendation and administration rates.

### 4.4 Communication and relationship with the general population

The next aspect is improving the awareness among the general population that pharmacists can vaccinate. Social media gained a growing role in providing personal health information and advice, including flu: a recent study delving the Twitter conversations around flu and vaccines showed that the public messaging exhibited a narrow scope, and broader topics should include the promotion of community, pharmacy-led vaccination (among others).^36^ With this background, holding public campaigns and posting the results of patient satisfaction surveys on social media platforms might help to increase the level of knowledge of the vaccinating pharmacist across the population. Novel pilot digital initiative to educate and engage the general population received positive feedback and registered improvement in the influenza vaccine uptake,^37^ inspiring to explore new approaches.

Findings from MacDougall et al. found that the focus group of the study had a concern about pharmacists managing adverse events requiring immediate attention.^38^Perhaps, increasing the public's awareness on the training pharmacists receive can help overcome such concerns.

## 5 Conclusion

Today, vaccinating pharmacists in Europe are continuing gaining recognition of their crucial role, and the subsequent benefits derived from their broader involvement in the healthcare system. Their acceptance was boosted in the post-pandemic phase, where they have played a critical and indispensable role in COVID-19 vaccination efforts, contributing significantly to successful immunization campaigns worldwide. As recently highlighted by the 2023 International Pharmaceutical Federation (FIP) publication “Pandemic preparedness, response and recovery: Lessons learnt for global pharmacy”,^39^ the pharmacy profession is appreciated for vaccine distribution, vaccine education and counseling, outreach to underserved communities, vaccine monitoring and adverse event reporting, and collaboration and interprofessional communication. We conclude that further efforts are still needed in each country to fulfil an adequate recognition of the profession. Strengthening the relations with policy makers/politicians to facilitate and reinforce the new role, generating more real-world evidence supporting the active role/benefits of pharmacists as vaccinators, improving the public awareness that pharmacists can vaccinate and enhancing the confidence, knowledge and skill set of pharmacists could be the key areas of improvement.

## Consent for publication

Not applicable.

## Ethics approval and consent to participate

This paper summarizes the opinion of the experts that participated in an advisory board meeting organized by the pharmaceutical company Viatris. The meeting was conducted in accordance with national and international laws on healthcare professional engagement by pharma company. The discussion did not include collection or analysis of any personal or medical data; thus no prior EC or IRB review was requested. The meeting and the publication process of the experts' opinion were conducted in line with the applicable policies and procedures.

## Funding

Viatris sponsored this study.

## CRediT authorship contribution statement

**Marleen Haems:** Writing – review & editing, Writing – original draft, Validation, Project administration, Methodology, Formal analysis, Conceptualization. **Mauro Lanzilotto:** Writing – review & editing, Writing – original draft, Validation, Supervision, Methodology, Investigation, Formal analysis, Data curation, Conceptualization. **Andrea Mandelli:** Writing – review & editing, Writing – original draft, Validation, Supervision, Project administration, Methodology, Investigation, Formal analysis, Data curation, Conceptualization. **Hélder Mota-Filipe:** Writing – review & editing, Writing – original draft, Validation, Supervision, Project administration, Methodology, Investigation, Formal analysis, Data curation, Conceptualization. **Ema Paulino:** Writing – review & editing, Writing – original draft, Validation, Supervision, Project administration, Methodology, Investigation, Formal analysis, Data curation, Conceptualization. **Beata Plewka:** Writing – review & editing, Writing – original draft, Validation, Supervision, Project administration, Methodology, Investigation, Formal analysis, Data curation, Conceptualization. **Olivier Rozaire:** Writing – review & editing, Writing – original draft, Validation, Supervision, Project administration, Methodology, Investigation, Formal analysis, Data curation, Conceptualization. **Jens Zeiger:** Writing – review & editing, Writing – original draft, Validation, Supervision, Project administration, Methodology, Investigation, Formal analysis, Data curation, Conceptualization.

## Declaration of competing interest

All the authors took part in the advisory board meeting conducted by Viatris. Rozaire Olivier is a paid consultant for Sanofi, Pfizer, Viatris, GSK and received speaker honoraria from Sanofi, Pfizer, Viatris, GSK, Moderna. Ema Paulino received speaker honoraria from Angelini, Bayer, Novo Nordisk, and Sanofi. Authors have no other competing interests to declare.

## Data Availability

The datasets used and/or analysed during the current study are available from the corresponding author on reasonable request.

## References

[1] Ortiz de Lejarazu-Leonardo R., Montomoli E., Wojcik R. (2021). Estimation of reduction in influenza vaccine effectiveness due to egg-adaptation changes-systematic. Literat Rev Expert Consen Vacc (Basel).

[2] Rajaram S., Wojcik R., Moore C. (2020). The impact of candidate influenza virus and egg-based manufacture on vaccine effectiveness: literature review and expert consensus. Vaccine.

[3] Olson S.M., Newhams M.M., Halasa N.B. (2022). Vaccine effectiveness against life-threatening influenza illness in US children. Clin Infect Dis.

[4] Ferdinands J.M., Thompson M.G., Blanton L., Spencer S., Grant L., Fry A.M. (2021). Does influenza vaccination attenuate the severity of breakthrough infections? A narrative review and recommendations for further research. Vaccine.

[5] Van Ranst M., Zollner Y., Schelling J., Palache B. (2023). The burden of seasonal influenza: improving vaccination coverage to mitigate morbidity and its impact on healthcare systems. Expert Rev Vaccines.

[6] Paudyal V., Fialova D., Henman M.C. (2021). Pharmacists’ involvement in COVID-19 vaccination across Europe: a situational analysis of current practice and policy. Int J Clin Pharmacol.

[7] Moniz E. (2020).

[8] Pharmacists E.C. (2023). Position Paper.

[9] Jacinto I.P., Costa S., Horta M.R. (2015). Serviço de vacinação nas farmácias portuguesas. Rev Portuguesa De Farmacoterap.

[10] Kanerva R. (2023).

[11] Papastergiou J., Folkins C., Li W., Zervas J. (2014). Community pharmacist-administered influenza immunization improves patient access to vaccination. Can Pharm J (Ott).

[12] L'Assurance Maladie (2024).

[13] Anderson C., Thornley T. (2014). “It’s easier in pharmacy”: why some patients prefer to pay for flu jabs rather than use the National Health Service. BMC Health Serv Res.

[14] Todd A., Copeland A., Husband A., Kasim A., Bambra C. (2015). Access all areas? An area-level analysis of accessibility to general practice and community pharmacy services in England by urbanity and social deprivation. BMJ Open.

[15] Warner J.G., Portlock J., Smith J., Rutter P. (2013). Increasing seasonal influenza vaccination uptake using community pharmacies: experience from the Isle of Wight, England. Int J Pharm Pract.

[16] Czech M., Balcerzak M., Antczak A. (2020). Flu vaccinations in pharmacies-a review of Pharmacists fighting pandemics and infectious diseases. Int J Environ Res Public Health.

[17] Burson R.C., Buttenheim A.M., Armstrong A., Feemster K.A. (2016). Community pharmacies as sites of adult vaccination: a systematic review. Hum Vaccin Immunother.

[18] Isenor J.E., Edwards N.T., Alia T.A. (2016). Impact of pharmacists as immunizers on vaccination rates: a systematic review and meta-analysis. Vaccine.

[19] Rahim M.H.A., Dom S.H.M., Hamzah M.S.R., Azman S.H., Zaharuddin Z., Fahrni M.L. (2024). Impact of pharmacist interventions on immunisation uptake: a systematic review and meta-analysis. J Pharm Policy Pract.

[20] Eurostat (2023).

[21] Piraux A., Cavillon M., Ramond-Roquin A., Faure S. (2022). Assessment of satisfaction with pharmacist-administered COVID-19 vaccinations in France: PharmaCoVax. Vaccines (Basel).

[22] Arcane Research (2024).

[23] Direção-Geral da Saúde (2024).

[24] dos Farmacêuticos Ordem (2024).

[25] Instituto Nacional de Estatistica (2023). Pharmacies and mobile medicine depots (No.) by Geographic localization (NUTS - 2013) and Type of local pharmaceutical unit. Annual.

[26] Portugal.Gov.PT (2023).

[27] International Pharmaceutical Federation (2016).

[28] The ABDA – Federal Union of German Associations of Pharmacists (2022).

[29] Narodowy Fundusz Zdrowia (2024).

[30] Antczak A., Balcerzak M., Byliniak M. (2020).

[31] Haems M. (2024).

[32] Kirkdale C.L., Nebout G., Taitel M. (2017). Implementation of flu vaccination in community pharmacies: understanding the barriers and enablers. Ann Pharm Fr.

[33] O’Reilly B., Isenor J., Bowles S. (2017). Expanding Pharmacists’ scope of practice to include immunization in Nova Scotia. Health Reform Observ - Observat des Réform Santé.

[34] O’Reilly D.J., Blackhouse G., Burns S. (2018). Economic analysis of pharmacist-administered influenza vaccines in Ontario, Canada. Clinicoecon Outcomes Res.

[35] Sayyed S.A., Kinny F.A., Sharkas A.R. (2024). Vaccination training for pharmacy undergraduates as a compulsory part of the curriculum?-a multicentric observation. Pharmacy (Basel).

[36] Ng Q.X., Ng C.X., Ong C., Lee D.Y.X., Liew T.M. (2023). Examining Public Messaging on Influenza Vaccine over Social Media: Unsupervised Deep Learning of 235,261 Twitter Posts from 2017 to 2023. Vaccines (Basel).

[37] Dale L.P., White L., Mitchell M., Faulkner G. (2019). Smartphone app uses loyalty point incentives and push notifications to encourage influenza vaccine uptake. Vaccine.

[38] MacDougall D., Halperin B.A., Isenor J. (2016). Routine immunization of adults by pharmacists: attitudes and beliefs of the Canadian public and health care providers. Hum Vaccin Immunother.

[39] International Pharmaceutical Federation (2023).

